# Transcriptome analysis reveals the mechanism of chronic heat stress on meat quality of broilers

**DOI:** 10.1186/s40104-022-00759-3

**Published:** 2022-09-19

**Authors:** Zhen Liu, Yingsen Liu, Tong Xing, Jiaolong Li, Lin Zhang, Yun Jiang, Feng Gao

**Affiliations:** 1 College of Animal Science and Technology, Key Laboratory of Animal Origin Food Production and Safety Guarantee of Jiangsu Province, Jiangsu Collaborative Innovation Center of Meat Production and Processing, Quality and Safety Control, Nanjing Agricultural University, No. 1 Weigang, Weigang 210095 Nanjing, China; 2 Institute of Agri-Products Processing, Jiangsu Academy of Agricultural Sciences, Nanjing, 210014 People’s Republic of China; 3 School of Food Science and Pharmaceutical Engineering, Nanjing Normal University, Nanjing, 210023 People’s Republic of China

**Keywords:** Chronic heat stress, Meat quality, Transcriptome

## Abstract

**Background:**

Chronic heat stress has a negative impact on poultry meat quality. Although this has been extensively investigated, previous studies have primarily focused on metabolic alterations and oxidative stress in the pectoralis major (PM) muscle under chronic heat stress, and not all of the underlying molecular mechanisms are completely understood.

**Methods:**

A total of 144 male Arbor Acres broilers (28 d old) were randomly allocated into 3 treatment groups: (1) the normal control (NC) group, with broilers raised at 22 °C and fed a basal diet; (2) the heat stress (HS) group, with birds raised at 32 °C and fed a basal diet; and (3) the pair-fed (PF) group, with birds raised at 22 °C and fed the amount of feed equal to the feed consumed on the previous day by the HS group. The experiment lasted for 14 d.

**Results:**

Chronic heat stress decreased the average daily feed intake and average daily gain, increased feed:gain ratio (*P* < 0.05); and increased drip loss, cooking loss, shear force, hardness, and decreased pH, redness (a^*^); and springiness of PM muscle (*P* < 0.05). Furthermore, chronic heat stress decreased muscle fiber density, increased connective tissue, and led to intracellular vacuolation. The transcriptome analyses indicated that the effect of chronic heat stress on meat quality was not only related to metabolism and oxidative stress, but also to signal transduction, immune system, transport and catabolism, cell growth and death, and muscle structure.

**Conclusions:**

Chronic heat stress has a negative impact on the growth performance, meat quality, and the PM muscle structure of broilers. Transcriptome analysis revealed a comprehensive understanding of the mechanism of the chronic heat stress-induced deterioration of broiler meat quality at the transcriptional level.

**Supplementary Information:**

The online version contains supplementary material available at 10.1186/s40104-022-00759-3.

## Introduction

Poultry meat has highly digestible proteins and low-fat content [1]. Modern broilers have a faster growth rate as a result of directional breeding in the past decades. Faster growth rate means greater metabolic activity and higher body heat production. Broilers are more susceptible to undesired high temperatures due to their thermoregulatory system (covered feathers and lack of sweat gland), and global warming renders broilers especially vulnerable to heat stress [2, 3].

Heat stress can cause sharp changes in the physiology and metabolism of birds, which negatively affect a variety of production parameters including feed intake and conversion efficiency, growth, egg production and quality, carcass traits, immune function, meat production, and quality [2, 4, 5]. In our previous study, we studied the metabolome changes caused by chronic heat stress and found that it caused a negative energy balance in broilers and rendering them unable to effectively oxidize fat for energy production, and instead resorting to protein decomposition [6]. Subsequently, our research team found that chronic heat stress impaired meat quality by altering redox status, energy metabolism, amino acid transport, glycolysis, intramuscular fat deposition, and protein synthesis [7, 8]. Although the negative impact of chronic heat stress on meat quality has been intensively studied, previous studies have primarily focused on metabolic changes and oxidative stress in the pectoralis major (PM) muscle under chronic heat stress, and the underlying molecular mechanisms are not completely understood.

The transcriptome is the complete set of transcripts and their quantity in cell or tissue. It can quantify the changing expression levels of each transcript during development and under different conditions [9]. Transcriptome analysis has been widely used to study the molecular mechanisms of tissue damage or metabolic changes caused by environmental stress and it can provide a definitive view of the role of the pathways and genes involved in stress [10, 11]. Therefore, in this study, the transcriptome analyses of broiler PM muscle were performed to identify the differentially expressed genes in broilers experiencing chronic heat stress and to identify the potential signal mechanisms involved. The results could comprehensively reveal the mechanism of inferior meat quality induced by chronic heat stress.

## Materials and methods

### Animals and experimental design

One hundred and forty-four chickens at 28 days of age (1460 ± 20 g) were randomly divided into three environmental chambers (Kooland Technology Co., Ltd., Beijing, China): normal control group (NC; 22 °C, ad libitum access to feed), heat stress group (HS; 32 °C, ad libitum access to feed) and pair-fed group (PF; 22 °C, fed the amount of feed equal to the feed intake of the previous day by the HS group). Each environmental chamber included six replicates (cages) (length × width × height: 1.2 m × 0.8 m × 0.45 m), and each cage included eight chickens. All birds had free access to water and received a common basal feed. The feed formula is shown in Table 1. The feed was provided in pellet form. The experiment lasted for 14 d.

**Table 1:** The compositions and nutrient levels of basal diet

**Items**	**Content**
Ingredients, %	
Corn	59.37
Soybean meal	31.90
Soybean oil	5.00
Limestone	1.23
Dicalcium phosphate	1.50
*L*-lysine	0.11
*DL*-methionine	0.27
Salt	0.30
Vitamin premix^a^	0.03
Mineral premix^b^	0.20
70% Choline chloride	0.09
Calculated nutrients, %	
Metabolizable energy, kcal/kg	3,100
Crude protein	19.00
Calcium	0.90
Total phosphorus	0.56
Available phosphorus	0.35
Lysine	1.00
Methionine	0.46
Methionine + cystine	0.80
Threonine	0.60
Tryptophan	0.20

^a^Vitamin premix provided per kilogram of diet: Vitamin A, 12,000 IU; Vitamin D_3_, 2500 IU; Vitamin E, 20 IU; menadione, 1.3 mg; thiamin, 2.21 mg; riboflavin, 7.8 mg; nicotinamide, 40 mg; calcium pantothenate, 16.5 mg; pyridoxine HCl, 4 mg; biotin, 0.04 mg; folic acid, 1.2 mg; Vitamin B_12_, 0.015 mg

^b^Mineral premix provided per kilogram of diet: iron, 80 mg; copper, 8.0 mg; manganese, 110 mg, zinc 65 mg; iodine, 1.1 mg; selenium, 0.3 mg

Daily feed intake was measured at 06:00 every day. The live weights of broilers were recorded on the 7^th^ and 14^th^ d of the experiment. Average daily gain (ADG), average daily feed intake (ADFI), and feed:gain ratio (F:G) were calculated at the end of the experiment.

### Infrared thermal image analysis

**Fig. 1:**
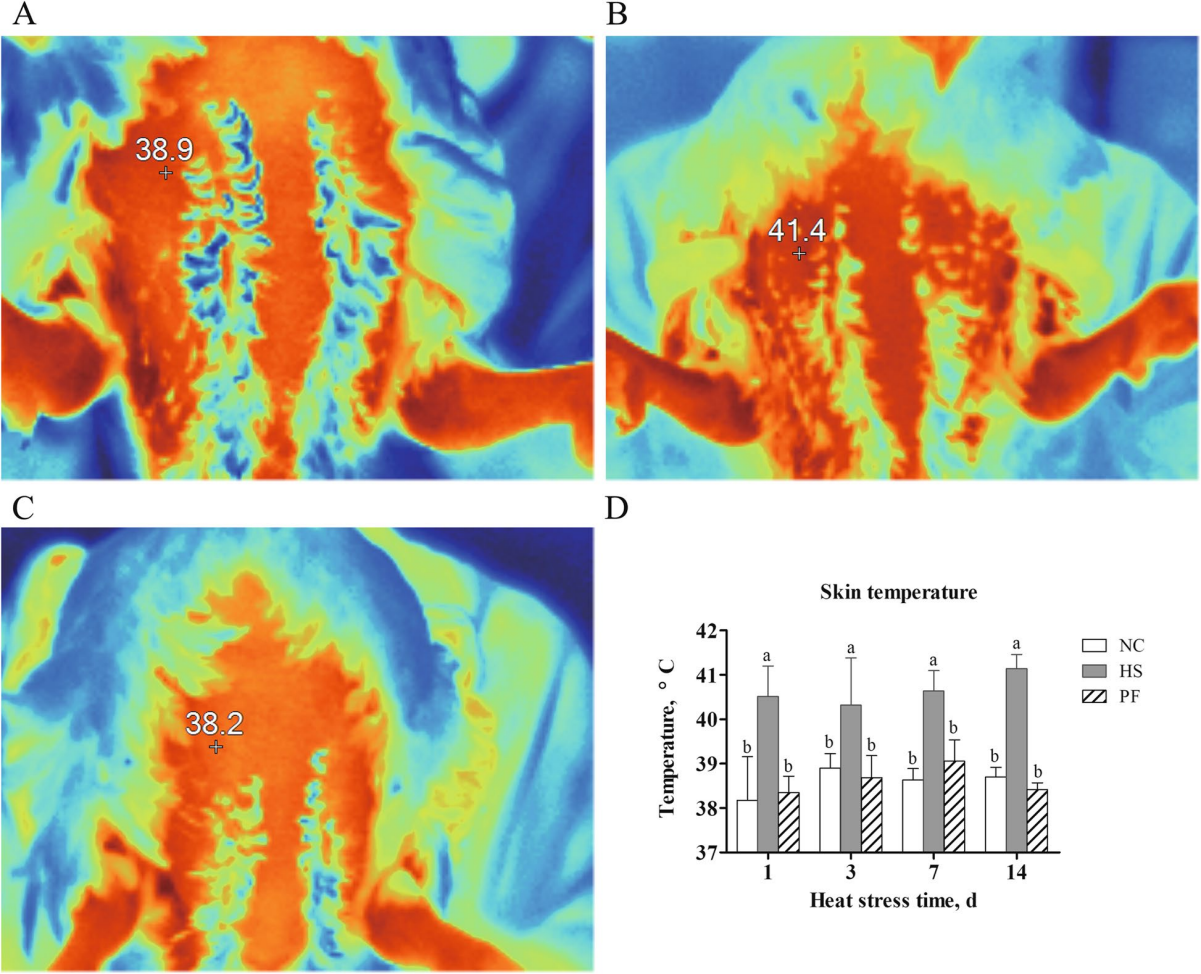
The body surface temperature of broilers. **A**, **B**, **C **The Infrared thermal image (After 14 d of heat stress) from the NC, HS and PF groups. **D** Body surface temperature of broilers in three groups in different times. The result represents the mean value ± standard deviation (SD) (*n* = 6). NC, normal control group; HS, heat stress group; PF, pair-fed group.^a,b^Different letters represent significant differences (*P* < 0.05)

At the 1^st^, 3^rd^, 7^th^, and 14^th^ d of the experiment, the body surface temperature (BST) of the birds was measured at 10:00 by using a Fluke Ti400 thermal imaging camera (Fluke Corporation, Everett, WA, USA) (Fig. 1A, B, and C). The BST of each chicken was presented as the average temperature of 6 points of measurements.

### Sample collection

At the 7^th^ (35 days of age) and 14^th^ (42 days of age) d of the experiment, 12 chickens (2 chickens per cage) were selected, electrically stunned, and slaughtered. The entire right PM muscle was stripped for meat quality determination. A sample of muscle (0.5 cm × 0.5 cm × 2 cm) was collected and fixed with 4% paraformaldehyde fixative solution. Additional PM muscle samples were collected in 3 cryovials and stored in liquid nitrogen.

### Measurement of meat quality

#### pH

A pH/ORP meter (HI9125, HANNA Instrument, Padova, Italy) was used to measure the muscle pH at 45 min and 24 h postmortem. The results for each meat sample are the average of 3 measurements at different locations.

#### Meat color

A chroma meter (CR410, Konica Minolta Sensing Inc., Osaka, Japan) was used to measure meat color at 24 h postmortem. The results for each meat sample are the average of 3 measurements at different locations.

#### Drip loss

The drip losses were measured as previously described [6]. Briefly, PM muscle samples (1 cm × 1 cm × 3 cm) were placed into a valve bag stored at 4 °C for 24 h, the samples were wiped and weighed (initial weight and final weight). Then, the percentage of lost weight was calculated.

#### Cooking loss

At 24 h postmortem, PM muscle samples (3 cm × 5 cm × 5 cm) were weighed and placed into valve bags and cooked until the central temperature reached 70 °C in a 72 °C water bath. When the samples were chilled to room temperature, they were wiped and reweighed. Then the percentage of lost weight was calculated.

#### Shear force

The samples after cooking loss determination were cut into 3 strips (1 cm × 1 cm × 3 cm) parallel to the muscle fibers. Then, the shear force was measured 3 times using a digital meat tenderizer (C1LM3, Northeast Agricultural University, Harbin, China). The results for each meat sample are the average of 3 measurements at different locations.

### Texture profile analysis

A cylindrical sample from cooked meat samples was taken with a sampler and then the textural characteristics were assessed by using a TMS-PRO texture analyzer (TA-XT plus, Stable Microsystem Ltd., Surrey, UK). The texture test parameters were as previously described [12].

### Hematoxylin and eosin staining

After dehydrated in a graded series of ethanol, paraformaldehyde-fixed muscle samples were embedded in paraffin and cut into 6 µm thick sections, and stained with hematoxylin and eosin. Images were captured using a light microscope (Olympus BX50 microscope, Tokyo, Japan).

### RNA extraction, sequencing and analysis

A total of 6 broilers in each group, were mixed in pairs to make 3 samples. Then, total RNA was extracted with 1 mL TRIzol reagent (Invitrogen, Carlsbad, CA, USA) according to the manufacturer's instructions. RNA quality was assessed using an Agilent 2100 Bioanalyzer (Agilent Technologies, Palo Alto, CA, USA). Sequencing libraries were constructed using an RNA sample preparation kit (Illumina, San Diego, CA, USA) according to the manufacturer's instructions. Then, the libraries were sequenced on the Illumina Hiseq Novaseq6000 Platform.

The fastp (version 0.18.0) was used to obtain high-quality, clean reads. The rRNA mapped reads were removed by using the Bowtie2 (version 2.2.8) tool. The software Stringtie and HISAT2 were used to align the clean reads to the reference genome.

The fragments per kilobase of transcript per million mapped fragments (FPKM) of each transcription region were calculated by using the RSEM software to quantify its expression abundance and variations. The R package gmodels [13] was used for principal component analysis (PCA).

DESeq2 software was used for differential expression analysis between two groups, and the genes with* P* < 0.05 and an absolute fold change >1.5 were considered differentially expressed. Then differentially expressed genes (DEGs) were carried on the GO functional and KEGG pathway enrichment analyses by using GOseq [14] and KOBAS [15], respectively. The *P*-values (*P* < 0.05) were used to determine the significantly enriched GO terms and KEGG pathways.

### Real-time PCR analysis

Twelve genes were selected for qRT-PCR analysis to verify the reliability of RNA sequencing results (RNA-Seq). Specific primer pairs were designed by using the Primer3 software [16] (Table 2). Reverse transcription was performed by using HiScript III RT Super Mix (Vazyme Biotech Co., Ltd, Nanjing, China), according to the manufacturer's instructions. Real-time qRT-PCR was carried out on an ABI QuantStudio™ 5 (Applied Biosystems, Waltham, MA, USA) using ChamQ Universal SYBR qPCR Master Mix (Vazyme Biotech Co., Ltd, Nanjing, China), according to the manufacturer's instructions. The expressions of relative genes were calculated by using the 2^−ΔΔCt^ method and β-actin was selected as reference gene for normalization.

**Table 2:** Primer sequences for real-time-quantitative-PCR analysis

Gene	GenBank number	Primer sequence (5′ to 3′)	Tm, °C	Product size, bp
*ACACA*	NM_205505.2	AACGAGTCGGGCTACTACCT	60.03	119
		ATCAGCATCCCGTGAAGTGG	60.11	
*LPL*	NM_205282.2	TTGAAGACCCGTGCTCAGAT	59.02	249
		ATCTTTCTCCCACTGCAGCT	59.01	
*PPARG*	NM_001397666.1	GAATGCCACAAGCGGAGAAGGAG	63.87	88
		*GCTCGCAGATCAGCAGATTCAGG*	63.72	
*SLC2A14*	NM_205511.2	TCCAGGCCTTCTACAACAGG	59.02	200
		CCCAGCAAAAGCCAAGACAT	59.03	
*PK*	NM_205469.2	GAACTGCGATGAGAATGTGC	57.55	105
		ACCAGCAAGGAAATGAGACC	57.79	
*LDHA*	NM_205284.2	ACTTGGTCCAACGCAATGTC	59.05	168
		TGCAGCCACTACCGATAACA	59.10	
*LDHB*	NM_204177.3	TGCTGAGCTCTGTGAGACAA	58.96	182
		TCAGCTGAGCCACTTCATCA	59.02	
*MYH1E*	NM_001397409.1	GAGAAGAGCAAGCAGAGCCAGATG	63.51	114
		CAACCTTGACACGAGGATAGCACAG	63.56	
*CNN2*	NM_001142256.2	GAAGATCAACCGCTCGGCACTG	64.01	139
		CTGTGTCAGGTTCCCACTCTCAAAG	63.20	
*TMOD1*	NM_205027.3	GTCTGGCATCCTGGCTCTTGTTG	63.83	81
		GGCTGGCTCTGGTTGTCGATTC	63.74	
*PRKCQ*	XM_040658916.1	ATGGTGCCCTGACACTTTGT	59.81	72
		TCACTCTCAACTGGTGTGCC	59.89	
*TGFB1*	NM_001318456.1	TGGATCCACGAACCCAAAGG	59.96	233
		CAGGCACGGACCACCATATT	60.11	
*TLR4*	NM_001030693.2	CATCCCAACCCAACCACAGTAGC	63.55	119
		CCACTGAGCAGCACCAATGAGTAG	63.46	
β-actin	NM_205518.2	ACGTCGCACTGGATTTCGAG	60.73	282
		TGTCAGCAATGCCAGGGTAC	60.32	

### Statistical analysis

One-way ANOVA was used to analyze the data using SPSS Statistics (version 20.0; SPSS Inc., Chicago, IL, USA). The results are presented as the mean ± standard deviation (SD), and the significant difference was set at *P* < 0.05. The data of growth performance were analyzed with the cage as the experimental replicate and other indices were analyzed by the mean of two sampled chickens per cage as a replicate (*n* = 6).

## Results

### Skin temperature

As shown in Fig. 1, the BST of the NC and PF groups was stably maintained at 38—39 °C, whereas the BST of the HS group was over 40 °C, significantly higher than the NC and PF groups (*P* < 0.05).

### Growth performance

Compared with the NC group, heat stress decreased the ADG and ADFI and increased the F:G ratio (*P* < 0.05, Table 3). Although the HS and PF groups had the same feed intake, the F:G ratio was higher in the HS group (*P* < 0.05, Table 3), the ADG of the PF group was significantly lower than that of the NC group after 14 d of the experiment (*P* < 0.05, Table 3). No significant difference was observed in the F:G ratio between NC and PF groups.

**Table 3:** Effects of chronic heat stress on the growth performance of broilers

Items	Treatments		
	NC	HS	PF
After 7 d of heat stress			
ADG, g	89.49 ± 7.09^a^	58.82 ± 10.93^b^	82.13 ± 9.91^a^
ADFI, g	150.93 ± 13.66^a^	127.22 ± 12.51^b^	134.36 ± 13.87^b^
F:G	1.69 ± 0.05^b^	2.21 ± 0.25^a^	1.64 ± 0.09^b^
After 14 d of heat stress			
ADG, g	90.55 ± 6.95^a^	49.80 ± 13.16^c^	68.6 ± 14.79^b^
ADFI, g	165.18 ± 11.51^a^	127.15 ± 15.86^b^	127.09 ± 17.5^b^
F:G	1.83 ± 0.10^b^	2.65 ± 0.36^a^	1.89 ± 0.18^b^

The result represents the mean value ± standard deviation (SD) (*n* = 6). *NC* normal control group, *HS* the heat stress group, *PF* the pair-fed group. *ADG* average daily gain, *ADFI* average daily feed intake, *F:G* feed:gain ratio

^a-c^Different letters represent significant differences (*P* < 0.05)

### Meat quality

At the 7^th^ d of the experiment, the pH_24h_ of PM muscle in the HS group was lower than that in the NC and PF groups (*P* < 0.05, Table 4). The drip loss and shear force value of the PM muscle in the HS group were higher than those in the NC and PF groups (*P* < 0.05, Table 4). At the 14^th^ d of the experiment, the pH_24h_ and a^*^ values of PM muscle in the HS group were lower; while the drip loss, cooking loss, and shear force were higher than those in the NC and PF groups (*P* < 0.05, Table 4).

**Table 4:** Effects of chronic heat stress on the meat qualities of pectoralis major (PM) muscle in broiler chickens

Items	Treatments		
	NC	HS	PF
After 7 d of heat stress			
pH_45min_^1^	6.28 ± 0.10	6.24 ± 0.18	6.28 ± 0.17
pH_24h_^2^	5.86 ± 0.04^a^	5.74 ± 0.02^b^	5.83 ± 0.09^a^
Lightness (L*)	42.58 ± 0.64	43.42 ± 1.58	43.79 ± 1.21
Redness (a*)	1.56 ± 0.20	1.51 ± 0.29	1.44 ± 0.18
Yellowness (b*)	3.98 ± 0.10	3.85 ± 0.49	4.35 ± 0.58
Drip loss, %	2.08 ± 0.14^b^	2.39 ± 0.12^a^	1.97 ± 0.05^b^
Cooking loss, %	12.10 ± 1.36	12.17 ± 0.95	12.87 ± 2.44
Shear force, N	15.96 ± 2.02^b^	19.56 ± 0.81^a^	12.79 ± 1.86^c^
After 14 d of heat stress			
pH_45min_	6.28 ± 0.13	6.37 ± 0.09	6.25 ± 0.11
pH_24h_	5.98 ± 0.04^a^	5.92 ± 0.06^b^	5.98 ± 0.03^a^
Lightness (L*)	43.55 ± 0.76	45.08 ± 0.78	43.4 ± 1.08
Redness (a*)	1.84 ± 0.31^a^	0.86 ± 0.15^b^	1.68 ± 0.23^a^
Yellowness (b*)	3.52 ± 0.49	3.67 ± 0.41	4.19 ± 0.61
Drip loss, %	1.82 ± 0.10^b^	2.16 ± 0.18^a^	1.93 ± 0.11^b^
Cooking loss, %	15.00 ± 0.71^b^	17.59 ± 0.47^a^	14.70 ± 0.83^b^
Shear force, N	18.76 ± 0.93^b^	20.88 ± 1.29^a^	18.49 ± 1.13^b^

The result represents the mean value ± standard deviation (SD) (*n* = 6). NC, normal control group; HS, heat stress group; PF, pair-fed group. ^1^pH_45min_, pH at 45 min post mortem; ^2^pH_24h_, pH at 24 h post mortem

^a-c^Different letters represent significant differences (*P* < 0.05)

### Textural properties

As shown in Table 5, At the 7^th^ d of the experiment, the PM muscle from the HS group had higher hardness and lower cohesiveness than those in the NC and PF groups (*P* < 0.05). Furthermore, the springiness of the PM muscle in the HS group was declined compared with that in the NC group (*P* < 0.05). At the 14^th^ d of the experiment, the hardness of the PM muscle from the HS group increased, while springiness decreased (*P* < 0.05).

**Table 5:** Effects of chronic heat stress on the textural properties of pectoralis major (PM) muscle in broiler chickens

Items	Treatments		
	NC	HS	PF
After 7 d of heat stress			
Hardness, N	26.11 ± 3.5^b^	31.39 ± 2.17^a^	26.15 ± 1.13^b^
Cohesiveness, -	0.45 ± 0.05^a^	0.41 ± 0.05^b^	0.45 ± 0.06^a^
Springiness, mm	2.62 ± 0.17^a^	2.35 ± 0.21^b^	2.40 ± 0.16^b^
Gumminess, N	13.11 ± 1.58	15.38 ± 2.81	12.83 ± 3.55
Chewiness, mJ	34.08 ± 4.44	37.71 ± 6.59	31.14 ± 6.71
After 14 d of heat stress			
Hardness, N	23.99 ± 3.52^b^	29.53 ± 1.87^a^	24.50 ± 3.48^b^
Cohesiveness, -	0.41 ± 0.02	0.40 ± 0.03	0.41 ± 0.02
Springiness, mm	3.13 ± 0.26^a^	2.58 ± 0.18^b^	3.19 ± 0.24^a^
Gumminess, N	11.88 ± 0.90	12.59 ± 2.43	12.60 ± 3.15
Chewiness, mJ	35.84 ± 3.25	33.71 ± 3.73	33.71 ± 3.94

The result represents the mean value ± standard deviation (SD) (*n* = 6). *NC* normal control group, *HS* heat stress group, *PF* pair-fed group

^a,b^Different letters represent significant differences (*P* < 0.05)

### Muscle structure

**Fig. 2:**
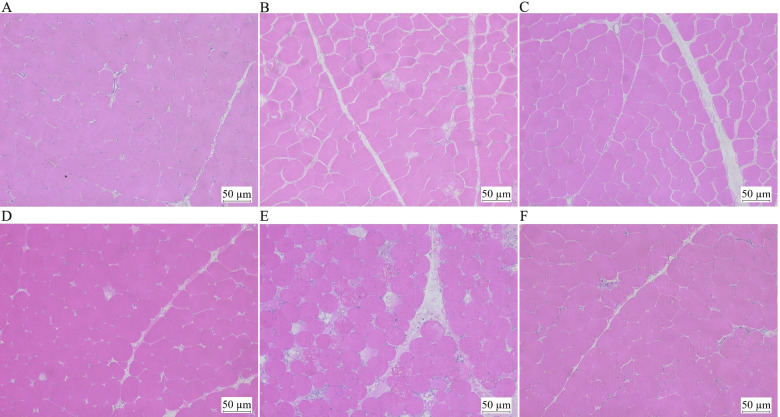
Hematoxylin and eosin staining of pectoralis major (PM) muscle sections. **A**, **B**, **C** The HE staining of the PM muscle (After 7 d of heat stress) from the NC, HS and PF groups. **D**, **E**, **F** The HE staining of the PM muscle (After 14 d of heat stress) from the NC, HS and PF groups. NC, normal control group; HS, heat stress group; PF, pair-fed group

The morphology of PM muscle in broilers is shown in Fig. 2. At the 7^th^ d of the experiment, PM muscle fibers from the HS group were scattered, the connective tissue increased, and vacuoles appeared in some cells compared with the NC and PF groups. At the 14^th^ d of the experiment, PM muscle damage in the HS group was further aggravated. At both time points, the morphology of the muscle fibers in the NC and PF groups were relatively intact and there was no damage. Heat stress had no significant effect on the fiber diameter of the PM muscle. Compared with the NC group, the muscle fiber density of the PM muscle in the HS and PF groups showed a decreasing trend (Table 6).

**Table 6:** Effects of chronic heat stress on the textural properties of pectoralis major (PM) muscle in broiler chickens

Items	Treatments		
	NC	HS	PF
After 7 d of heat stress			
Density, N/μm^2^	1042.58 ± 94.97	969.58 ± 46.02	994.02 ± 60.20
Diameter, μm	29.60 ± 0.76	29.12 ± 1.51	29.00 ± 2.81
After 14 d of heat stress			
Density, N/μm^2^	842.74 ± 76.54	793.33 ± 75.16	744.10 ± 95.45
Diameter, μm	35.66 ± 1.83	34.59 ± 1.26	34.60 ± 2.10

The result represents the mean value ± standard deviation (SD) (*n* = 6). *NC* normal control group, *HS* heat stress group, *PF* pair-fed group

### RNA sequencing and differential expression analysis

**Fig. 3:**
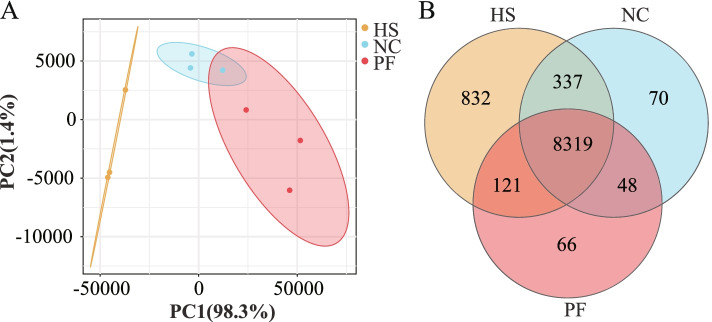
Principal component analysis (PCA) and Wayne (VENN) analysis. **A** Principal component analysis (PCA) of the samples in three groups. **B** Venn diagram of the identified genes in pectoralis major (PM) muscle of three groups. NC, normal control group; HS, heat stress group; PF, pair-fed group

**Fig. 4:**
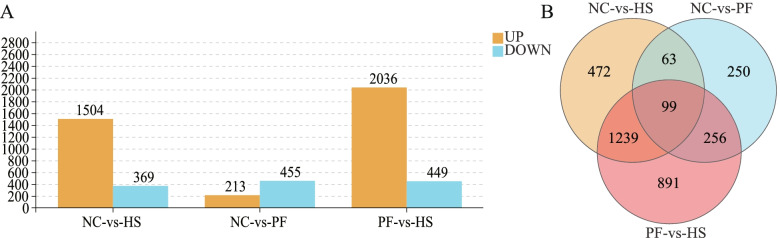
Differentially expressed genes (DEGs) in HS vs. NC, HS vs. PF and NC vs. PF. **A** the numbers of DEGs in pectoralis major (PM) muscle after heat stress. **B** the Venn diagram of DEGs in pectoralis major (PM) muscle after heat stress. NC, normal control group; HS, heat stress group; PF, pair-fed group

From the PCA result (Fig. 3A) we found that the HS group had a unique transcriptional profile, and heat stress was the principal component (98.3%). In the Venn diagram (Fig. 3B), 8774, 9609, and 8554 genes were identified in the NC, HS, and PF groups, respectively. Compared with the NC group, 1873 differently expressed genes (DEGs) were identified in PM muscle from the HS group, including 1054 up-regulated genes and 369 down-regulated genes. Compared with the PF group, 2485 DEGs were identified in PM muscle from the HS group, including 2036 up-regulated genes and 449 down-regulated genes. Compared with the NC group, 668 DEGs were identified in PM muscle from the PF group, among which 213 genes were up-regulated and 445 genes down-regulated in the PF groups (Fig. 4A). Based on the Venn diagram analysis of DEGs (Fig. 4B), 472 unique DEGs were identified in HS vs. NC, 891 unique DEGs were identified in HS vs. PF and 250 unique DEGs were identified in NC vs. PF. We also found 1338 DEGs shared by HS vs. NC and HS vs. PF; and 99 DEGs shared by HS vs. NC, HS vs. PF, and NC vs. PF. To exclude the factor of feed intake and further investigated the effects of heat stress, we selected 1239 DEGs as the target DEGs, which were shared by HS vs. NC and HS vs. PF after removing the DEGs of NC vs. PF (Fig. 4B). Among the target DEGs, 1034 genes were up-regulated and 205 genes were down-regulated in the HS group compared with NC and PF groups. From these DEGs, we identified some genes that were potentially related to meat quality and listed in Additional file 1: Table S1.

Heat stress significantly up-regulated genes related to lipid metabolism and synthesis processes such as acyl-CoA synthetase family members (*ACSS1A*, *ACSS1B*, *ACSS2*, *AACS*), acetyl-CoA carboxylase alpha (*ACACA*), carnitine palmitoyl transferase 2 (*CPT2*), acyl-CoA thioesterase 9 (*ACOT9*), diacylglycerol kinase zeta (*DGKZ*), glycerol-3-phosphate acyltransferase 3 (*GPAT3*), 3-hydroxyacyl-CoA dehydratase 3 (*HACD3*), lipoprotein lipase (*LPL*), lipase A (*LIPA*), lipin 2 (*LPIN2*), palmitoyl-protein thioesterase 1 (*PPT1*), and peroxisome proliferator-activated receptor gamma (*PPARG*). Glucose metabolic processes related genes such as fructose-bisphosphatase 2 (*FBP2*), bisphosphoglycerate mutase (*BPGM*), glucose-6-phosphate isomerase (*GPI*), glycogen synthase (*GYS1*), pyruvate dehydrogenase kinase 4 (*PDK4*), lactate dehydrogenase A (*LDHA*), probable D-lactate dehydrogenase mitochondrial isoform X2 (*LDHD*), pyruvate dehydrogenase phosphatase catalytic subunit 1 (*PDP1*), fructose-2,6-biphosphatase 3 (*PFKFB3*), phosphoglucomutase 1 (*PGM1*), phosphoglycerate mutase 1 (*PGAM1*), phosphoglucomutase 2 like 1 (*PGM2L1*), phosphoglycerate kinase 2 (*PGK2*), pyruvate kinase (*PKM*), succinate-CoA ligase ADP-forming beta subunit (*SUCLA2*) and triosephosphate isomerase 1 (*TPI1*) were down-regulated in PM muscle from HS group compared with that from the NC and PF groups. Aconitase 1 (*ACO1*), aldolase fructose-bisphosphate C (*ALDOC*), aconitate decarboxylase 1 (*ACOD1*), enolase family members (*ENO1*, and *ENO2*), fructose-bisphosphatase 1 (*FBP1*), aldolase fructose-bisphosphate C (*GPD1L2*), lactate dehydrogenase B (*LDHB*), phosphogluconate dehydrogenase (*PGD*), phosphoglucomutase 2 (*PGM2*), and solute carrier family 2 members (*SLC2A14*, and *SLC2A6*) were up-regulated in birds from the HS group. In the HS group, muscle structure related genes such as actin alpha1 (*ACTA1*), myotubularin related protein 7 (*MTMR7*), myosin family members (*MYH1E*, *MYH1F*, *MYL1*, *MYLK2*, *MYO18B*, *MYOM1*), tropomodulin 4 (*TMOD4*), troponinI 2 (*TNNI2*), troponin I3 (*TNNT3*), were down-regulated, while actin beta (*ACTB*), actinin alpha1 (*ACTN1*), actin filament associated protein 1 (*AFAP1*), ARP2 actin related protein 2 family members (*ACTR2*, *ACTR3*), anillin actin binding protein (*ANLN*), actin related protein 2/3 complex subunit family members (*ARPC1B*, *ARPC2*, *ARPC3*, *ARPC5*, *ARPC5L*), coactosin like F-actin binding protein 1 (*COTL1*), calponin 2 (*CNN2*), destrin actin depolymerizing factor (*DSTN*), Fascin actin-bundling protein 1(*FSCN1*), leiomodin 2 (*LMOD2*), myosin family members (*MYH1B*, *MYO1E*, MYO1F, *MYO3AL*, *MYO7A*), spire type actin nucleation factor 1 (*SPIRE1*), tropomodulin 1 (*TMOD1*) were up-regulated. Compared with the NC and PF groups, all genes related to apoptosis listed in Table 2 were up-regulated in birds from the HS group, except sestrin 1 (*SESN1*), solute carrier family 25 member 4 (*SLC25A4*), apoptosis-associated tyrosine kinase (*AATK*), heat shock protein 90 alpha family class B member 1 (*HSP90AB1*), mitofusin 2 (*MFN2*), and programmed cell death 2-like (*PDCD2L*).

### Functional annotation and pathway enrichment analysis of the target DEGs

**Fig. 5:**
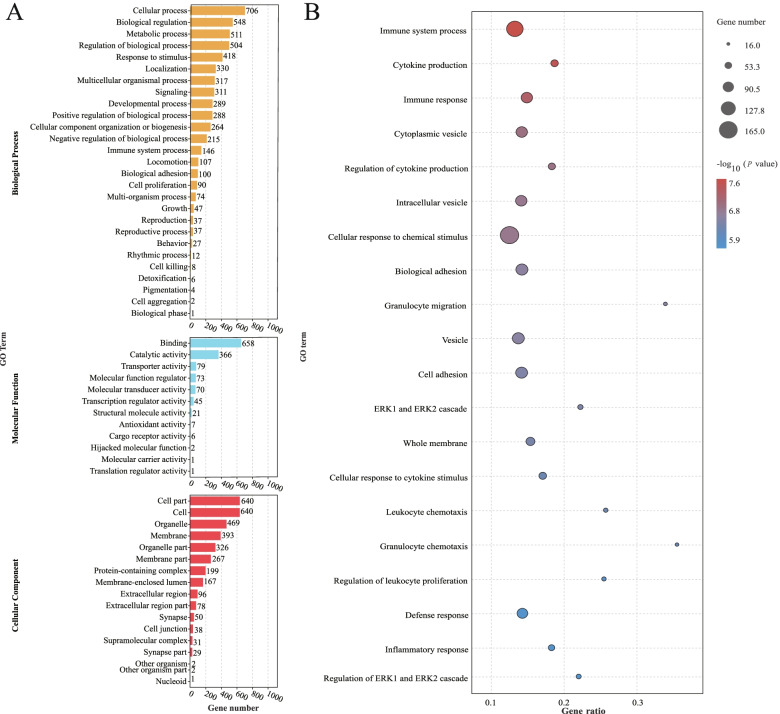
GO terms enrichment of target DEGs. **A** The GO enrichment of the target DEGs. **B** Top 20 GO enrichment for the target DEGs. NC, normal control group; HS, heat stress group; PF, pair-fed group

In order to identify the biological function of the target DEGs, they were classified according to GO functional enrichment (Fig. 5A). The most enriched terms in Biological Process were cellular process, biological regulation, metabolic process, regulation of biological process, and response to stimulus metabolic process. In Molecular Function the most enriched terms were binding and catalytic activity. In Cellular Component the most enriched terms were cell part, cell, organelle, membrane. Additionally, immune system process, cytokine production, cell adhesion, ERK1 and ERK2 cascade, cellular response to chemical stimulus, inflammatory response were significantly enriched (Fig. 5B).

**Fig. 6:**
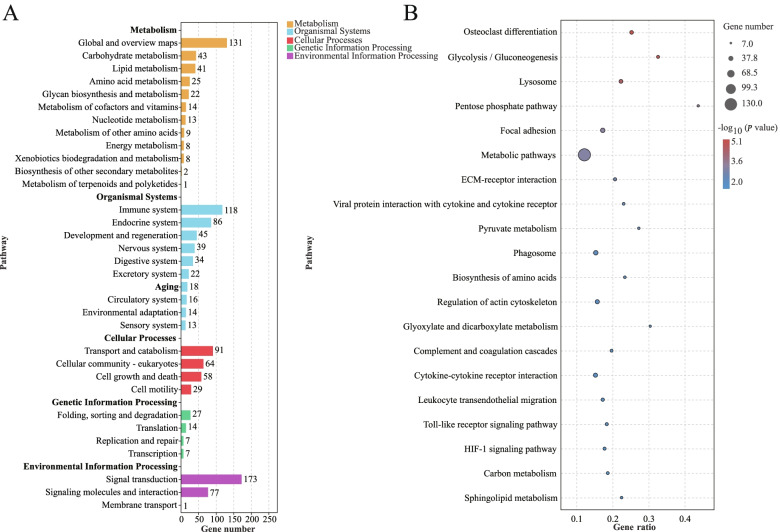
KEGG pathways enrichment of target DEGs. **A** The KEGG enrichment of the target DEGs. **B** Top 20 of KEGG pathways enrichment for the target DEGs. NC, normal control group; HS, heat stress group; PF, pair-fed group

Furthermore, KEGG pathway analysis was used to identify the biological pathways of the target DEGs (Fig. 6A and B). The KEGG annotation results revealed that the three most enriched metabolic pathways were carbohydrate metabolism, lipid metabolism and amino acid metabolism. The immune system, endocrine system and nervous system were the three most enriched organismal systems. The top three enriched cellular processes pathways were transport and catabolism, cellular community-eukaryotes, and cell growth and death. The three most enriched genetic information processing pathways were folding, sorting and degradation, translation, replication and repair. The top three enriched environmental information processing pathways were signal transduction, signaling molecules and interaction, and membrane transport.

From the top 20 significantly enriched KEGG pathways (Fig. 6B) we found that metabolic pathways were the most enriched, including glycolysis/gluconeogenesis, pentose phosphate pathway, pyruvate metabolism, biosynthesis of amino acids, glyoxylate and dicarboxylate metabolism, carbon metabolism and sphingolipid metabolism. In addition, lysosome, phagosome, regulation of actin cytoskeleton, HIF-1 signaling pathway, cytokine-cytokine receptor interaction, and toll-like receptor signaling pathway were also significantly enriched.

### qRT-PCR validation of differentially expressed genes

**Fig. 7:**
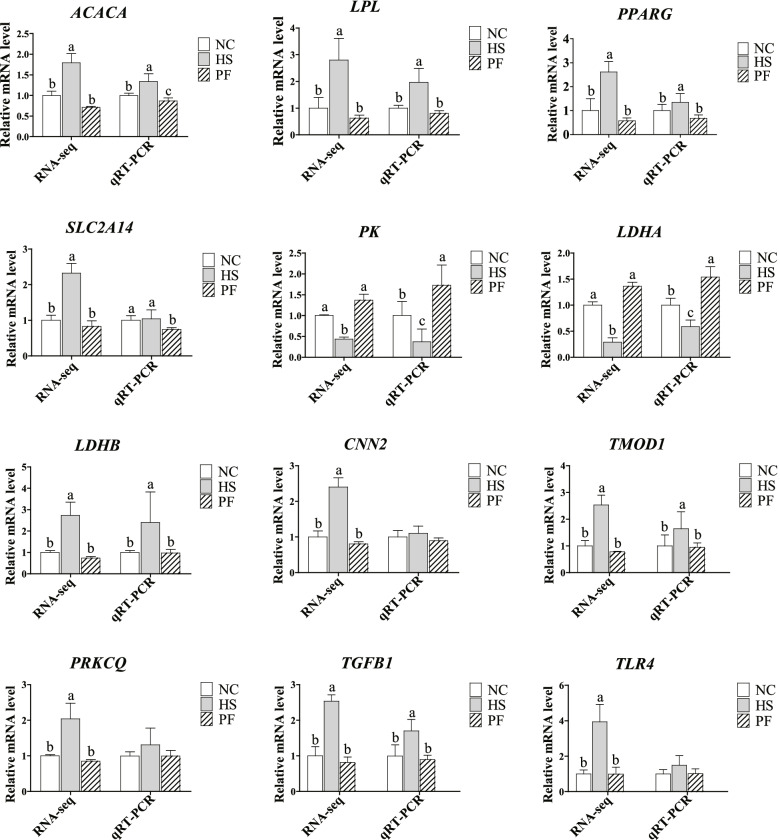
qRT-PCR of DEGs compared with RNAseq. The data presented as the relative expression of both RPKM (*n* = 3) and qRT-PCR (*n* = 6) and data that are shown as the mean ± standard deviation (SD) values. NC, normal control group; HS, the heat stress group; PF, the pair-fed group. ^a,b^Different letters represent significant differences (*P* < 0.05)

The relative mRNA level trend of the genes were consistent with the RNA sequencing data (Fig. 7), indicating that the transcriptomic of the current study were reliable.

## Discussion

Broiler is a homeothermic animal that maintains a relatively constant body temperature through the thermoregulatory system by adjusting behavioral and physiological responses to increase or decrease body heat loss. When the ambient temperature is raised beyond the ability of animals to dissipate heat, the animals experience heat stress [17]. Body surface temperature is considered as an ideal physiological indicator of heat stress in animals [2, 18–20]. In the current research, BST was used to evaluate the heat stress model. Heat stress significantly elevated the BST from the first day of the experiment, indicating that the broilers were in a state of heat stress.

Previous studies reported that chronic heat stress reduces growth performance by impairing the gut integrity and appetite of broilers [2, 4]. The current study showed that chronic heat stress significantly decreased the ADFI and ADG, and increased the F:G. Although the HS and the PF groups had the same feed intake, the ADG and feed conversion ratio of birds from the HS group were lower than those from the PF group, indicating that the decrease of growth performance is caused by a combination of high temperature and feed intake decrease. In line with previous reports [6, 21]. Our previous research reported that chronic heat stress significantly increased L^*^ values, drip loss, and cooking loss and decreased the pH of broiler PM muscle [7]. In the current study, we found that chronic heat stress increased the drip loss, cooking loss, and shear force value and decreased the pH_24h_ and a^*^ value of the PM muscle. It value of the PM muscle. It has been reported that all texture properties (hardness, elasticity, cohesion, and chewiness) increased after a cyclic high-temperature treatment [22]. Similarly, we found that chronic heat stress resulted in an increase in hardness, but the springiness of the PM muscle was decreased.

In the current study, the transcriptome of PM muscle was used to explore the mechanisms of inferior meat quality induced by chronic heat stress. The PCA result demonstrated that heat stress was the main factor responsible for the difference in the transcriptional profiles of the HS group (98.3%), indicating that chronic heat stress was the major factor causing the deterioration of growth performance and meat quality. Confirmed the previous hypothesis that heat stress reduces growth performance has another mechanism, besides the reduction in feed intake. In order to investigate the effects of chronic heat stress and eliminate the influence of feed intake, we deleted the DEGs between the NC and PF groups. Then, selected 1239 DEGs as the target DEGs from those remaining genes in the HS groups, which were significantly compared with the NC and PF groups. The target genes were related to metabolic process, response to stimulus, catalytic activity, cell part, cell growth and death, HIF-1 signaling pathway, lysosome, regulation of actin cytoskeleton, and sphingolipid metabolism. Among the target DEGs, the genes involved in cell part, membrane, and sphingolipid metabolism are related to cell membrane integrity and apoptosis under heat stress [23]. Evidence shows that damage to muscle membranes may lead to a reduction in meat quality [24], and we observed damaged muscle cells and loose muscle fibers in the HS group. This could be one of the reasons for the increased drip loss and cooking loss observed in this experiment.

Heat stress also affects the redox status and induces oxidative stress. The cytochrome P450 superfamily members (CYPs) are a group of heme-containing proteins that catalyze the activation of molecular oxygen, resulting in the generation of reactive oxygen species (ROS) [25, 26]. In tissues, excessive ROS accumulation can cause oxidative stress [27]. Due to their ability to induce ROS production, the CYPs could be considered an effective RNA biomarker for evaluating oxidative stress. Wang et al. reported that 42 d of NH_3_ exposure induced up-regulation of the CYPS and oxidative stress in the jejunum of broilers [28]. In this current study, RNA-seq analysis showed that the mRNA expression of CYPS (*CYP1C1*, *CYP2J21*, *CYP2J23*, *CYP39A1*, *CYP4A22*) were up-regulated in birds from the HS group (Additional file 1: Table S1). This is in line with our previous finding that chronic heat stress generates excess ROS, resulting to oxidative stress [7]. Noeman et al. showed that ROS induces respiratory chain oxidative damage which in turn could increase ROS generation and a vicious cycle ensues [29]. In the current study, the damage of the muscle fiber caused by heat stress was aggravated with time, demonstrating the occurrence of oxidative stress in the PM muscle induced by heat stress.

Chronic heat stress has been shown to cause lipid metabolism changes [30]. In the present study, some genes associated with fat synthesis were up-regulated, including *ACACA*, *GPAT3*, and *PPARG*. This might lead to the deposition of intramuscular fat, which is consistent with the previous study [7]. Zhao et al. indicated that heat stress significantly reduced the oxidation of fatty acids in skeletal muscle [31]. In this study, although the genes associated with lipolysis such as *LPIN2*, *LPL*, and *LIPA* were up-regulated while the carnitine palmitoyl transferase1 (*CPT1*), one of the rate-limiting enzymes of fat oxidation, was down-regulated in HS group compared with the NC (log_2_(FC): −0.35) and PF (log_2_(FC): −1.04) groups. These results may be the reason for the decrease in beta-oxidation in heat-stressed birds.

Heat stress also affects energy metabolism. In this study, we found that the expression of isocitrate dehydrogenase 3 (NAD ( +)) beta, a rate-limiting enzyme of the tricarboxylic acid cycle, was down-regulated of broilers experienced heat stress, which might result in a reduction in ATP production. These findings were consistent with previous results which showed that chronic heat stress damaged the mitochondrial structure and affected muscle energy metabolism [7, 32]. At the same time, we found that the glucose transporters *SLC2A14*, *SLC2A6,* and ATPase family members were up-regulated (Additional file 1: Table S1). This may be a negative feedback regulation of energy reduction. A series of reports have demonstrated that the rate of muscle glycogen breakdown is faster in heat-stressed chickens than that in normal birds [33, 34]. Similarly, we found that the expression of *GYS1* in muscle was down-regulated in birds in the HS groups. These results imply that chronic heat stress affects meat quality by affecting muscle energy metabolism.

Several studies have reported that heat stress causes muscle damage. Chronic heat stress causes histopathological damage in broilers [35]. Sula et al. found that heat stress induces multifocal myocyte degeneration, necrosis, and ultimately rhabdomyolysis in the muscles of lambs [36]. Heat stress induces red cusk-eel skeletal muscle atrophy at the molecular level [37]. In the current study, the expression of the actin family members and related protein members, except *ACTA1*, were up-regulated in the HS group. The expression of myosin family members *MYH1E*, *MYH1F*, *MYL1*, *MYLK2*, and *MYO18B* were down-regulated, while *MYH1B*, *MYO1E*, *MYO1F*, *MYO3AL*, and *MYO7A* were up-regulated in the HS group. Moreover, the expression of *TNNT3* and *TNNI2* in the HS group were decreased. Actin, myosin, and troponin are structural proteins of muscle cells and they play a pivotal role in the mechanism of muscular contraction [38, 39]. The molecular architecture of the sarcomere as defined by the myosin filaments and their S-1 and S-2 units, the interaction with the actin filaments, and the boundaries formed by the Z-disks, will subsequently influence basic meat quality traits such as tenderness and water-holding capacity [40]. GO and KEGG analysis showed that regulation of actin cytoskeleton organization and ECM-receptor interaction were significantly enriched. In the present study, histological observations showed that some muscle fibers were degraded, and connective tissue increased in the HS group, demonstrating that chronic heat stress causes muscle damage. This may be related to the increased drip loss, cooking loss, shear force, and hardness observed in the HS group.

Several studies have demonstrated that heat stress affects apoptosis. Heat stress activates the FasL/Fas and TNF-α systems and induces apoptosis of follicular cells in laying hens [41]. Heat exposure induces apoptosis in mouse gastrocnemius muscle cells [42]. In addition, heat stress increased the apoptosis of porcine semitendinosus skeletal muscle cells [43]. Heat stress caused endoplasmic reticulum stress, and oxidative stress activated C/EBP homologous protein (*CHOP*) and subsequently activated cell apoptosis [44, 45]. Chen et al. found that heat stress reduced the viability and induced apoptosis of myoblasts [46]. In this study, many apoptosis-related genes including TNF receptor superfamily member (TNFRSF), transforming growth factor beta 1 (*TGFB1*), v-myc avian myelocytomatosis viral oncogene homolog (*MYC*), Janus kinase 1 (*JAK1*), and programmed cell death 1 (*PDCD1*) were up-regulated. In the present study, the histological observation found that vacuoles were observed inside the cell and some muscle fibers were degraded in the HS groups. These results demonstrate that chronic heat stress causes cell apoptosis. Our previous study showed that oxidative stress led to apoptosis and autophagy and resulted in a decline in meat quality by decreasing muscle pH_24h_ and increasing shear force value [47]. This may be one of the reasons for the increased shear force in this study.

## Conclusions

In conclusion heat stress has a negative impact on growth performance and meat quality. The transcriptome analyses indicated that the effect of chronic heat stress on meat quality was not only related to metabolism and oxidative stress, but also to signal transduction, immune system, transport and catabolism, cell growth and death and muscle structure. The results provide a comprehensive understanding of the mechanism of chronic heat stress-induced deterioration of broiler meat quality at the transcriptional level.

## Supplementary Information

**Additional file 1:** **Table S1. **List of some differentially expressed genes related to meat quality.

## Data Availability

All data generated or analyzed during this study are available from the corresponding authors on reasonable request.

## Abbreviations

NC: Normal control
HS: Heat stress
PF: Pair-fed
PM: Pectoralis major
ADG: Average daily gain
ADFI: Average daily feed intake
F:G: Feed:gain ratio
BST: Body surface temperature
