# Supplementary material for: Transcriptome analysis reveals the mechanism of chronic heat stress on meat quality of broilers
Source: J Anim Sci Biotechnol. 2022 Sep 19;13:110. doi: 10.1186/s40104-022-00759-3 (PMC9484139; doi:10.1186/s40104-022-00759-3)
Supplement: Supplementary file 1 — Additional file 1: Table S1. List of some differentially expressed genes related to meat quality. [file 40104_2022_759_MOESM1_ESM.docx]

**Additional file 1**

**Table S1** **Differentially expressed genes related to meat quality**

| Symbol | Description | log_2_(fc)^a^ | | *P* value | |
| --- | --- | --- | --- | --- | --- |
|  |  | NC vs. HS | PF vs. HS | NC vs. HS | PF vs. HS |
| *ACSS1* | acyl-CoA synthetase short-chain family member 1A | 0.90 | 1.44 | 0.05 | 0.01 |
| *AACS* | acetoacetyl-CoA synthetase | 0.90 | 1.35 | 0.04 | <0.01 |
| *ACACA* | acetyl-CoA carboxylase alpha | 0.84 | 1.33 | 0.02 | <0.01 |
| *ACOT9* | acyl-CoA thioesterase 9 | 0.73 | 1.31 | 0.02 | <0.01 |
| *ACSS1B* | acyl-CoA synthetase short-chain family member 1B | 1.87 | 2.19 | <0.01 | <0.01 |
| *ACSS2* | acyl-CoA synthetase short-chain family member 2 | 0.83 | 1.04 | 0.04 | 0.01 |
| *CPT2* | carnitine palmitoyl transferase 2 | 1.13 | 1.17 | <0.01 | 0.01 |
| *DGKZ* | diacylglycerol kinase zeta | 1.28 | 1.09 | 0.01 | <0.01 |
| *GPAT3* | glycerol-3-phosphate acyltransferase 3 | 1.09 | 1.45 | 0.01 | <0.01 |
| *HACD3* | 3-hydroxyacyl-CoA dehydratase 3 | 0.89 | 1.28 | 0.01 | <0.01 |
| *LIPA* | lipase A, lysosomal acid type | 1.65 | 2.11 | <0.01 | <0.01 |
| *LPIN2* | lipin 2 | 1.10 | 1.21 | <0.01 | 0.01 |
| *LPL* | lipoprotein lipase | 1.49 | 2.16 | <0.01 | <0.01 |
| *PPARG* | peroxisome proliferator-activated receptor gamma | 1.39 | 2.20 | 0.01 | <0.01 |
| *PPT1* | palmitoyl-protein thioesterase 1 | 1.02 | 1.31 | <0.01 | <0.01 |
| *BPGM* | bisphosphoglycerate mutase | -0.92 | -1.02 | <0.01 | <0.01 |
| *FBP2* | fructose-bisphosphatase 2 | -1.06 | -1.60 | <0.01 | <0.01 |
| *GPI* | glucose-6-phosphate isomerase | -0.83 | -0.94 | <0.01 | <0.01 |
| *GYS1* | glycogen [starch] synthase, muscle-like | -1.06 | -1.06 | <0.01 | <0.01 |
| *LDHA* | lactate dehydrogenase A | -1.77 | -2.22 | <0.01 | <0.01 |
| *LDHD* | probable D-lactate dehydrogenase, mitochondrial isoform X2 | -0.73 | -0.67 | <0.01 | <0.01 |
| *PDK4* | pyruvate dehydrogenase kinase 4 | -2.70 | -2.13 | 0.01 | <0.01 |
| *PDP1* | pyruvate dehyrogenase phosphatase catalytic subunit 1 | -0.83 | -1.03 | <0.01 | <0.01 |
| *PFKFB3* | 6-phosphofructo-2-kinase/fructose-2,6-biphosphatase 3 | -1.70 | -2.08 | <0.01 | <0.01 |
| *PGAM1* | phosphoglycerate mutase 1 | -1.42 | -1.73 | <0.01 | <0.01 |
| *PGK2* | phosphoglycerate kinase 2 | -1.33 | -1.59 | <0.01 | <0.01 |
| *PGM1* | phosphoglucomutase 1 | -0.92 | -1.07 | <0.01 | <0.01 |
| *PGM2L1* | phosphoglucomutase 2 like 1 | -0.78 | -0.88 | <0.01 | <0.01 |
| *PKM* | pyruvate kinase, liver and RBC | -1.20 | -1.66 | <0.01 | <0.01 |
| *SUCLA2* | succinate-CoA ligase ADP-forming beta subunit | -0.97 | -1.31 | <0.01 | <0.01 |
| *TPI1* | triosephosphate isomerase 1 | -1.31 | -1.6 | <0.01 | <0.01 |
| *ACO1* | aconitase 1, soluble | 1.02 | 1.30 | <0.01 | <0.01 |
| *ACOD1* | aconitate decarboxylase 1 | 2.62 | 2.19 | 0.01 | <0.01 |
| *ALDOC* | aldolase, fructose-bisphosphate C | 1.58 | 1.77 | <0.01 | <0.01 |
| *ENO1* | enolase 1 (alpha) | 1.16 | 1.34 | <0.01 | <0.01 |
| *ENO2* | enolase 2 | 2.77 | 2.82 | <0.01 | <0.01 |
| *FBP1* | fructose-bisphosphatase 1 | 1.63 | 2.05 | <0.01 | <0.01 |
| *GPD1L2* | glycerol-3-phosphate dehydrogenase 1-like 2 | 1.60 | 1.85 | <0.01 | <0.01 |
| *LDHB* | lactate dehydrogenase B | 1.45 | 1.88 | <0.01 | <0.01 |
| *PGD* | phosphogluconate dehydrogenase | 1.25 | 1.76 | <0.01 | <0.01 |
| *PGM2* | phosphoglucomutase 2 | 1.20 | 1.73 | 0.01 | <0.01 |
| *SLC2A14* | solute carrier family 2 member 14 | 1.22 | 1.49 | <0.01 | <0.01 |
| *SLC2A6* | solute carrier family 2 member 6 | 1.87 | 1.86 | <0.01 | <0.01 |
| *ACTA1* | actin alpha 1, skeletal muscle | -1.01 | -1.19 | <0.01 | <0.01 |
| *MTMR7* | myotubularin related protein 7 | -1.32 | -1.09 | <0.01 | <0.01 |
| *MYH1E* | myosin, heavy chain 1E, skeletal muscle | -1.29 | -1.33 | <0.01 | <0.01 |
| *MYH1F* | myosin, heavy chain 1F, skeletal muscle | -1.39 | -0.67 | <0.01 | <0.01 |
| *MYL1* | myosin, light chain 1, alkali; skeletal, fast | -0.95 | -0.96 | <0.01 | <0.01 |
| *MYLK2* | myosin light chain kinase 2 | -0.71 | -1.24 | <0.01 | <0.01 |
| *MYO18B* | myosin XVIIIB | -0.94 | -1.19 | <0.01 | <0.01 |
| *MYOM1* | myomesin 1 | -0.76 | -0.67 | <0.01 | <0.01 |
| *TMOD4* | tropomodulin 4 | -0.94 | -1.51 | <0.01 | <0.01 |
| *TNNI2* | troponin I2, fast skeletal type | -0.89 | -1.00 | <0.01 | <0.01 |
| *TNNT3* | troponin T3, fast skeletal type | -0.86 | -0.69 | <0.01 | <0.01 |
| *ACTB* | actin, beta | 0.99 | 1.12 | <0.01 | 0.01 |
| *ACTN1* | actinin, alpha 1 | 1.06 | 1.46 | <0.01 | <0.01 |
| *ACTR2* | ARP2 actin related protein2 homolog | 1.00 | 1.32 | <0.01 | <0.01 |
| *ACTR3* | ARP3 actin related protein3 homolog | 1.44 | 1.65 | <0.01 | <0.01 |
| *AFAP1* | actin filament associated protein 1 | 1.2 | 1.36 | 0.03 | 0.02 |
| *ANLN* | anillin actin binding protein | 1.78 | 2.21 | <0.01 | <0.01 |
| *ARPC1B* | actin related protein 2/3 complex subunit 1B | 1.34 | 1.51 | <0.01 | <0.01 |
| *ARPC2* | actin related protein 2/3 complex subunit 2 | 0.96 | 1.27 | <0.01 | <0.01 |
| *ARPC3* | actin related protein 2/3 complex subunit 3 | 1.44 | 1.47 | <0.01 | <0.01 |
| *ARPC5* | actin related protein 2/3 complex subunit 5 | 1.23 | 1.62 | <0.01 | <0.01 |
| *ARPC5L* | actin related protein 2/3 complex subunit 5 like | 3.35 | 2.44 | <0.01 | 0.02 |
| *CNN2* | calponin 2 | 1.27 | 1.57 | <0.01 | <0.01 |
| *COTL1* | coactosin like F-actin binding protein 1 | 1.26 | 1.36 | <0.01 | <0.01 |
| *DSTN* | destrin, actin depolymerizing factor | 0.98 | 1.28 | <0.01 | <0.01 |
| *FSCN1* | fascin actin-bundling protein 1 | 1.26 | 1.95 | 0.01 | <0.01 |
| *LMOD2* | leiomodin 2 | 2.36 | 2.33 | <0.01 | <0.01 |
| *MYH1B* | Gallus gallus myosin, heavy chain 1G, skeletal muscle | 2.04 | 3.03 | <0.01 | <0.01 |
| *MYO1E* | myosin IE | 1.34 | 1.56 | <0.01 | <0.01 |
| *MYO1F* | myosin IF | 1.95 | 2.17 | <0.01 | <0.01 |
| *MYO3AL* | myosin IIIA-like | 2.20 | 2.97 | <0.01 | <0.01 |
| *MYO7A* | myosin VIIA | 1.79 | 1.71 | <0.01 | 0.04 |
| *SPIRE1* | spire type actin nucleation factor 1 | 1.5 | 1.78 | 0.01 | 0.02 |
| *TMOD1* | tropomodulin 1 | 1.34 | 1.70 | <0.01 | <0.01 |
| *AATK* | apoptosis associated tyrosine kinase | -1.87 | -1.53 | <0.01 | <0.01 |
| *HSP90AB1* | heat shock protein 90 alpha family class B member 1 | -1.34 | -1.64 | <0.01 | <0.01 |
| *MFN2* | mitofusin 2 | -0.74 | -0.7 | <0.01 | <0.01 |
| *PDCD2L* | programmed cell death 2-like | -0.62 | -0.81 | 0.01 | <0.01 |
| *SESN1* | sestrin 1 | -2.11 | -2.22 | <0.01 | <0.01 |
| *SLC25A4* | solute carrier family 25 member 4 | -1.64 | -1.90 | <0.01 | <0.01 |
| *ATG4B* | autophagy related 4B cysteine peptidase | 1.07 | 1.53 | <0.01 | <0.01 |
| *BCL2A1* | BCL2 related protein A1 | 1.63 | 1.70 | <0.01 | 0.01 |
| *BCL2L10* | BCL2 like 10 | 0.99 | 1.41 | 0.04 | <0.01 |
| *BCL6* | B-cell CLL/lymphoma 6 | 1.17 | 1.56 | <0.01 | <0.01 |
| *CARD11* | caspase recruitment domain family member 11 | 0.97 | 1.37 | <0.01 | <0.01 |
| *CARD9* | caspase recruitment domain family member 9 | 2.24 | 2.07 | <0.01 | <0.01 |
| *CASP18* | initiator caspase | 1.36 | 1.33 | 0.02 | 0.03 |
| *CTSC* | cathepsin C | 1.54 | 1.89 | <0.01 | <0.01 |
| *CTSH* | cathepsin H | 1.49 | 1.39 | <0.01 | <0.01 |
| *CTSK* | cathepsin K | 1.40 | 1.82 | <0.01 | <0.01 |
| *CTSS* | cathepsin S | 1.56 | 1.80 | <0.01 | <0.01 |
| *CTSZ* | cathepsin Z | 1.09 | 1.39 | <0.01 | <0.01 |
| *HIF1A* | hypoxia inducible factor 1 subunit alpha | 0.84 | 1.23 | 0.05 | <0.01 |
| *IFNGR2* | interferon gamma receptor 2(interferon gamma transducer 1) | 0.91 | 1.48 | 0.01 | <0.01 |
| *ITPR2* | inositol 1,4,5-trisphosphate receptor type 2 | 1.01 | 1.20 | <0.01 | 0.01 |
| *JAK1* | Janus kinase 1 | 0.84 | 1.02 | 0.04 | <0.01 |
| *LOC378902* | Gallus death domain-containing tumor necrosis factor receptor super family member 23 | 1.48 | 1.46 | <0.01 | 0.01 |
| *MYC* | v-myc avian myelocytomatosis viral oncogene homolog | 1.52 | 1.81 | <0.01 | <0.01 |
| *PDCD1* | programmed cell death 1 | 2.17 | 1.95 | <0.01 | <0.01 |
| *PDCD1LG2* | programmed cell death 1 ligand 2 | 2.77 | 2.86 | <0.01 | <0.01 |
| *PERP1* | PERP1, TP53 apoptosis effector | 3.06 | 4.93 | <0.01 | <0.01 |
| *PIK3CB* | phosphatidylinositol-4,5-bisphosphate 3-kinase catalytic subunit beta | 1.79 | 1.73 | <0.01 | <0.01 |
| *PIK3CD* | phosphatidylinositol-4,5-bisphosphate 3-kinase catalytic subunit delta | 1.74 | 2.83 | <0.01 | <0.01 |
| *PRKCQ* | protein kinase C theta type | 1.03 | 1.26 | <0.01 | <0.01 |
| *SERPINB5* | serpin family B member 5 | 6.98 | 4.25 | 0.01 | 0.04 |
| *SKP2* | S-phase kinase associated protein 2 | 1.08 | 1.40 | <0.01 | <0.01 |
| *SLC25A5* | solute carrier family 25 member 5 | 0.79 | 1.35 | 0.04 | <0.01 |
| *SMAD3* | SMAD family member 3 | 1.12 | 1.39 | <0.01 | <0.01 |
| *STK4* | serine/threonine kinase 4 | 1.10 | 1.36 | <0.01 | <0.01 |
| *TGFB1* | transforming growth factor beta 1 | 1.34 | 1.64 | <0.01 | <0.01 |
| *TGFB2* | transforming growth factor beta 2 | 0.90 | 1.25 | 0.02 | 0.01 |
| *TGFBR2* | transforming growth factor beta receptor 2 | 0.91 | 0.97 | <0.01 | 0.03 |
| *TLR4* | toll like receptor 4 | 1.98 | 1.98 | <0.01 | <0.01 |
| *TNFRSF1A* | TNF receptor superfamily member 1A | 0.86 | 1.09 | 0.02 | <0.01 |
| *TNFRSF21* | TNF receptor superfamily member 21 | 1.12 | 1.19 | <0.01 | <0.01 |
| *ATP1A1* | ATPase Na+/K+ transporting subunit alpha 1 | 1.00 | 1.19 | 0.01 | <0.01 |
| *ATP2B1* | ATPase plasma membrane Ca2+ transporting 1 | 1.03 | 1.24 | 0.01 | 0.01 |
| *ATP6AP1L* | ATPase H+ transporting accessory protein 1 like | 2.78 | 3.97 | 0.04 | 0.02 |
| *ATP6AP2* | ATPase H+ transporting accessory protein 2 | 1.23 | 1.19 | <0.01 | 0.01 |
| *ATP6V0B* | ATPase H+ transporting V0 subunit b | 0.86 | 1.09 | 0.03 | <0.01 |
| *ATP6V0D1* | ATPase H+ transporting V0 subunit d1 | 0.83 | 1.05 | 0.01 | <0.01 |
| *ATP6V0D2* | ATPase H+ transporting V0 subunit d2 | 3.01 | 3.24 | <0.01 | <0.01 |
| *ATP6V1A* | ATPase H+ transporting V1 subunit A | 1.14 | 1.82 | <0.01 | <0.01 |
| *ATP6V1B2* | ATPase H+ transporting V1 subunit B2 | 1.12 | 1.25 | <0.01 | <0.01 |
| *ATP6V1H* | ATPase H+ transporting V1 subunit H | 1.06 | 1.16 | <0.01 | <0.01 |
| *CYP1C1* | cytochrome P450 family 1 subfamily C polypeptide 1 | 3.81 | 4.49 | <0.01 | <0.01 |
| *CYP2J21* | cytochrome P450, family 2 subfamily J polypeptide 21 | 1.19 | 1.42 | 0.02 | 0.01 |
| *CYP2J23* | cytochrome P450, family 2 subfamily J polypeptide 23 | 1.58 | 1.35 | <0.01 | 0.02 |
| *CYP39A1* | cytochrome P450 family 39 subfamily A member 1 | 1.41 | 1.39 | <0.01 | <0.01 |
| *CYP4A22* | cytochrome P450 family 4 subfamily A member 22 | 1.56 | 2.31 | 0.03 | <0.01 |

*NC*, normal control group; *HS*, heat stress group; *PF*, pair-fed group.

^a^log_2_(fc): log_2_(Fold change)
